# Grain boundary, electrical transport and thermoelectric properties of the ultra-high rGO amount of C12A7-rGO composites

**DOI:** 10.1016/j.heliyon.2024.e29619

**Published:** 2024-04-12

**Authors:** Keerati Maneesai, Montree Thongkam, Chaval Sriwong, Chesta Ruttanapun

**Affiliations:** a Smart Materials Research and Innovation Unit, School of Science, King Mongkut's Institute of Technology Ladkrabang, Chalongkrung Road, Ladkrabang, Bangkok, 10520, Thailand; b Department of Physics, School of Science, King Mongkut's Institute of Technology Ladkrabang, Chalongkrung Road, Ladkrabang, Bangkok, 10520, Thailand; c Department of Chemistry, School of Science, King Mongkut's Institute of Technology Ladkrabang, Chalongkrung Road, Ladkrabang, Bangkok, 10520, Thailand; d Thailand Center of Excellence in Physics, Ministry of Higher Education, Science, Research and Innovation, 328 Si Ayutthaya Road, Bangkok, 10400, Thailand

**Keywords:** C12A7 and ultra-high amount rGO composites, Thermoelectric properties, Electrical transport properties, Grain boundary, Agglomeration

## Abstract

The Ca_12_Al_14_O_33_ ceramic (C12A7) and reduced graphene oxide (rGO) composite which an ultra-high amount (i.e., 40, 50, 60, and 70 wt%) of rGO (ultra-high amount C12A7/rGO composite) were synthesized by a solid-state reaction process. After the hydraulic press, the heat treatment in the temperature range of 773 K under the argon environment had been performed with the composite pellets for 30 min. XRD results of the C12A7 and all the ultra-high amount C12A7/rGO composites indicated a pure phase of C12A7 ceramic. Raman spectra confirmed the existence of rGO content in all the ultra-high amount C12A7/rGO composites. Raman peaks also suggested reduction of the free $O_{2}^{2-}$ and $O_{2}^{-}$ ions from the framework of the ultra-high amount C12A7/rGO composites. SEM image presented the homogeneous grain boundary interface after the heat treatment at 773 K of the C12A7 wrapped by the rGO sheet, the agglomerated rGO sheet, and the rough interface stack of rGO sheets. UV-VIS spectroscopy presented the absorption behavior, direct energy gap, and indirect energy gap modifications of the ultra-high amount C12A7/rGO composites. Electrical conductivity of the ultra-high amount C12A7/rGO composites illustrated larger than 10^8^ times improvement with temperature independence. Range of −5 to −17 μV/K , temperature dependence, and increased with rGO content increasing Seebeck coefficient were reported. Thermal conductivity of the ultra-high amount C12A7/rGO composites was increased with the rGO content increasing. Both the Power factor (PF) and the figure of merit (ZT) of the ultra-high amount C12A7/rGO composites were temperature dependent and were increased with the rGO content increasing, within the range of 0.4 $\mu W/m.K^{2}$ of PF and the range of ${3x10}^{-4}$ of ZT, respectively. These experimental results verified grain boundary, modified energy band, electrical transport properties and thermoelectric properties of C12A7/rGO composites loading with ultra-high content rGO

## 1 Introduction

Dodecacalcium hepta aluminate or 12CaO·7Al_2_O_3_ (Ca_12_ Al_14_O_33_) or C_12_A_7_ or mayenite is an organic phase of calcium aluminate ceramic. The crystal structure of C_12_A_7_ is classified as a cubic symmetry structure which a space group of I-43d and a lattice parameter of 11.989 Å [1]. The crystal structure of C1_2_A_7_ exhibits a 3D framework of tetrahedra AlO_2_ interconnected with octahedral Ca^2+^. A unit cell of C1_2_A_7_ consists of 12 interconnected cages of the positive charge of [Ca_24_ Al_28_ O_64_]^4+^ framework equivalent with two free oxygen anions (O^2−^) distributed one per six cages. The free oxygen anions are loosely bound by electrostatic force from six calcium inside the cages. The high oxide conductivity of C12A7 is caused by these two oxygen ions [2].

Due to the abundance of natural raw elements, calcium, and aluminum oxides, insulating C12A7 mayenite promises a potential candidate for oxide ion conducting material applications such as solid oxide fuel cells (SOFC), oxygen sensors, supercapacitors, oxygen pumps, and batteries [2]. Substitution of the free oxygen anions with other anions such as OH^−^, H^−^, O^−^, O^2-^, F-, electron and others for specific functions had been reported. Substituted the free oxygen anions with electrons can occupy the empty space inside each cage forming electride materials which much higher electrical conductivity but do not improve oxide ion conductivity [3]. Another approach to enhance properties of C12A7 mayenite, the incorporation of insulating materials and rGO composites into network wrapping structure in 3D results in enormous improvements in both electrical conductivity and relative increase of ZT with respect to ZT of the initial materials [4]. However, the agglomeration of the second phase content in the low-amount rGO composites decreases the electrical conductivity due to the scattering point of the agglomerated rGO layer of the composites but increases oxygen vacancy due to additional thermal activation at the agglomerated rGO [5]. Several different behaviors in electrical transport properties and thermoelectric properties of pure insulating SrTiO_3_ (STO) after the addition of low-amount rGO of (1) rGO content≤0.7 wt% and (2) rGO content≥1 wt% had been reported [5]. Morphological results of the STO and rGO composites by SEM indicated no agglomeration of the rGO content≤0.7 wt% composites and some agglomeration at the grain boundary of the rGO content≥1 wt% composites. Mobility of the STO and rGO composites was significantly enhanced by the addition of rGO contents. Mobility of the rGO content≤0.7 wt% composites was greater than three times of magnitude in the initial pure STO sample. Mobility of the rGO content≥1 wt% composites was decreased from the mobility of the rGO content≤0.7 wt% composites. The concentration of the rGO content≤0.7 wt% composites was temperature independent and increased with the content of rGO increasing. The concentration of the rGO content≥1 wt% composites was greater than the concentration of the rGO content≤0.7 wt% composites, increased with the content of rGO increasing, and was slightly temperature dependent. The electrical conductivity of the rGO content≤0.7 wt% composites was temperature dependence and increased with the content of rGO increasing. The electrical conductivity of the rGO content≥1 wt% composites was also temperature dependent but decreased with the content of rGO increasing. The magnitude of Seebeck coefficient of both the rGO content≤0.7 wt% composites and the rGO content≥1 wt% composites were lower than the magnitude of Seebeck coefficient of the pure STO, temperature independence, and decreased with the content of rGO increasing. The thermal conductivity of both the rGO content≤0.7 wt% composites and the rGO content≥1 wt% composites were lower than the thermal conductivity of the pure STO and negative temperature dependence. The thermal conductivity of the rGO content≤0.7 wt% composites was decreased but the thermal conductivity of the rGO content≥1 wt% composites was increased with the content of rGO increasing.

Several different behaviors in electrical conductivity and thermoelectric properties of C12A7 and nano-carbon black (nCB) composites with (1) the nCB content 0 and 1 wt% composites and (2) the nCB content 3,5 and 10 wt% composites also had been reported by Rudradawong [6]. Electrical conductivity of both the nCB content 0 and 1 wt% composites and the nCB content 3,5 and 10 wt% composites were increased with the content of nCB increasing. Electrical conductivity of the nCB content 0 and 1 wt% composites was a little bit temperature dependence but electrical conductivity of the nCB content 3, 5 and 10 wt% composites was temperature independence. While Seebeck coefficient of the nCB content 0 and 1 wt% composites could not measure, the Seebeck coefficient of the nCB content 3, 5 and 10 wt% composites were temperature dependence and increased with the nCB content increasing. Thermal conductivity of the nCB content 0 and 1 wt% composites was constant but the thermal conductivity of the nCB content 3, 5 and 10 wt% composites was decreased with the nCB content increasing. Thermal conductivity of the nCB content 0 and 1 wt% composites was temperature dependence but the thermal conductivity of the nCB content 3, 5 and 10 wt% composites was temperature independence. Power factor (PF) and dimensionless figure of merit (ZT) of the nCB content 0 and 1 wt% composites were could not measure but the PF and the ZT of the nCB content 3, 5 and 10 wt% composites was temperature dependence and increased with the nCB content increasing. Previous research on C12A7/rGO composites with low rGO content has shown promising results, predicting improvements in electrical transport and thermoelectric properties within a specific limit. However, these benefits are limited by the rGO content. To capitalize on the high conductivity offered by rGO networks, this research explores C12A7/rGO composites loaded with ultra-high rGO content as an alternative approach. This research aims to investigate how C12A7/rGO composites loaded with ultra-high rGO approach affects the grain boundaries induced by the agglomerated rGO, the modified energy band structure, and ultimately, the electrical transport and thermoelectric properties of the composites.”

The experiment study of C12A7/rGO composite loading with ultra-high content of rGO had been performed. The following section details the synthesis methods of C12A7/rGO composites with varying rGO content, characterization techniques employed to analyze grain boundaries, energy bands and finally, the methods used to measure electrical transport and thermoelectric properties.

## 2 Experiments

### 2.1 Graphene oxide and reduced graphene oxide preparations

As reported by the previous research [7,8], graphene oxide (GO) was synthesized using a modified Hummers' method then followed by ultra-sonication of graphite oxide in absolute acetone media. In brief, about 3 g of graphite was mixed with about 9 g of KMnO4, then poured into a round bottom flask and cooled below 3 °C. After that, about 70 mL of H_2_SO_4_ was added gradually and stirred at 313 K for 30 min. Subsequently, about 135 mL of DI water was slowly added, and then heated at 368 K under stirring in oil bath for 60 min. After that, the reaction was stopped by adding 380 mL of DI water, followed by 15 mL of H_2_O_2_ and 15 mL of HCl solution (5 % v/v), respectively. Then, the suspended mixture was filtered and washed several times by DI water until the pH of filtered solution was about 7. The resulting solid paste product was dried in the oven at 338 K overnight, and the GO powder was obtained. To prepare the suspension of GO in acetone media, about 600 mg of the obtained GO powder was redisposed in absolute acetone (100 mL) under ultra-sonication for 90 min, followed by centrifugation. Finally, the dark brown supernatant of GO suspension was obtained. Reduced graphene oxide (rGO) using in this research was synthesized by modified chemical reduction method [9]. In brief, 100 mL of the obtained GO (2 mg/mL) suspension was mixed with 100 mL of DI water in a beaker, followed by addition of 1 mL of an ammonia and 0.1 mL of hydrazine hydrate solutions. Then, the mixture was heated and stirred at 368 K in oil bath for 45 min. During the reduction, the suspension color changed from brown to black. Finally, a stable aqueous suspension of rGO nanosheets was obtained.

### 2.2 C12A7 preparation

In preparation of C12A7, Calcium carbonate (CaCO_3_, 98 % purity Sigma Aldrich) and alumina powder (Al_2_O_3_, 98 % purity Sigma Aldrich) were used as starting materials. A ratio of 12:7 CaCO_3_ and Al_2_O_3_ powders were mixed for 24 h in a ball milling machine. Then, the mixed powder was calcined at 1623 K for 3 h under a natural air atmosphere.

### 2.3 C12A7 and ultra-high amount rGO composites and the composite pellet preparations

**Fig. 1:**
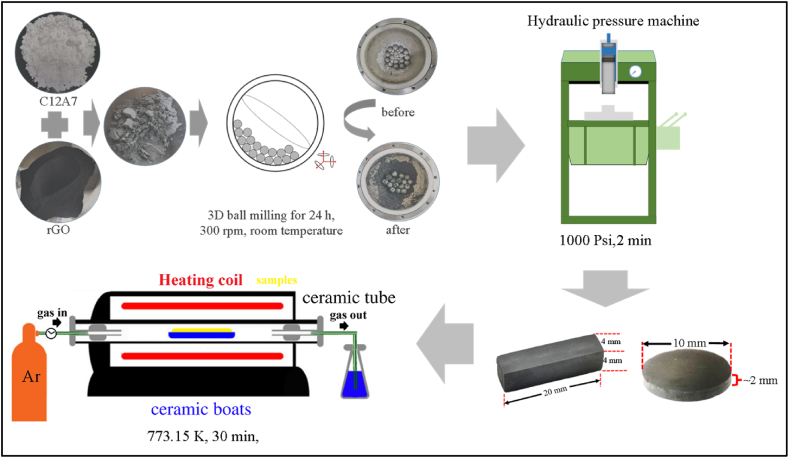
Schematic diagram of C12A7 and ultra-high amount rGO composites preparation.

The C12A7 and ultra-high amount rGO composite (C12A7/rGO composites) preparation as shown in Fig. 1, the obtained C12A7 powder was mixed with rGO for 24 h and 300 rpm in a 3D ball milling machine (Nagao system inc., 3D reactor). 3D mixing time and rpm variation affect particle size and homogenously of composites. Our 3D mixing condition was optimized to obtain a small particle size with a homogeneous structure of C12A7/rGO composites. The C12A7/rGO composites consist of 0, 40, 50, 60 and 70 % weight rGO (denoted by C12A7, C12A7+40 wt%rGO, C12A7+50 wt%rGO, C12A7+60 wt%rGO and C12A7+70 wt%rGO) respectively. According to the literature reviews, the excess limit of the low amount of loading rGO content is about 15 wt%. During our experiment, thermoelectric properties of the ultra-high amounts C12A7/rGO composite loading with 20 and 30 wt% of rGO content also were investigated. However, Seebeck coefficient and power factor of the sample with 20 and 30 wt% could not be measured. The mixed power was compressed under hydraulic uniaxial pressure of 5000 kPa at room temperature with a holding time of 2 min into rectangular rod with size of about 5 mm × 5 mm x 20 mm and circular pellet with diameter of 10 mm and high of about 2 mm. Then, the composite pellet was annealed in a tube furnace at 773 K for 30 min under argon atmosphere.

## 3 Results and discussion

### 3.1 Characterizations

The structure of C12A7, rGO and C12A7 and ultra-high amount rGO composites were investigated using X-ray diffractometry (XRD) with a 2θ scanning range from 10 to 80° and sample stepping interval of 0.02° in Rigaku, Miniflex Cu K-alpha radiation (CuKα) = 1.5406 Å). Thermogravimetric analysis (TGA) was performed in Netzsch STA 449F3 Jupiter to measure mass loss and rate of mass loss of the samples. Scanning Electron Microscope (SEM) and Energy Dispersive X-Ray Spectrometer: (EDS) were performed in FEI model Quanta 250 to investigate morphology and material compositions of the samples. UV-VIS was performed in Shimadzu model UV-2600 spectrometer to measure optical properties of the samples. Dispersive Raman spectroscopy was used to confirm vibrational characteristic of the samples in Thermo Fisher Scientific Inc. model DXR Smart Raman. Resistivity, electrical conductivity, carrier concentration, mobility and Hall coefficient were measured in Linseis model, Hall-Effect Characterization System HCS 1. Seebeck coefficient and power factor of the sample were measure in LINSEIS model LSR-3. Thermal conductivity of the sample was measured using In-house method based on Standard Test Method for Thermal Diffusivity by the Flash Method (ASTM E1461-13).

#### 3.1.1 X-Ray diffraction analysis

**Fig. 2:**
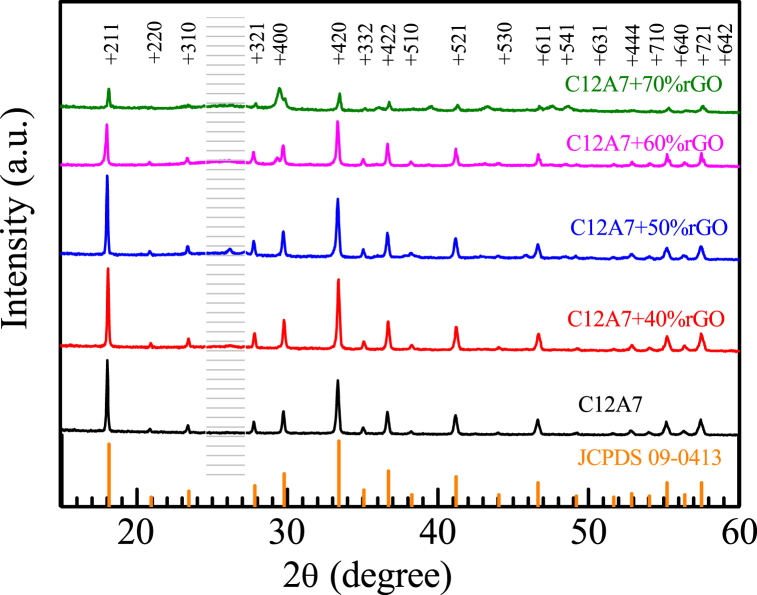
X-Ray diffraction pattern of C12A7 and C12A7/rGO composites.

The X-Ray diffraction pattern of C12A7 and ultra-high amount C12A7/rGO composites are shown in Fig. 2. The characteristic peak of C12A7 matched the JCPDs number 09–0413 standard peak pattern. This confirmed the successful synthesis of a C12A7 ceramic phase. The XRD patterns of all the ultra-high amount C12A7/rGO composite relating to the C12A7 sample. The highlight in Fig. 2 demonstrates very small broad peak corresponding to the plane of (002) with d spacing of 0.354 nm of rGO structure [7,8] at 2θ = 25.14°. The calculated crystallite size using Scherer's equation of C12A7, C12A7+40 wt%rGO, C12A7+50 wt%rGO, C12A7+60 wt%rGO and C12A7+70 wt%rGO are 48.7, 47.9, 43.6, 47.1, and 45.8 nn, respectively.

#### 3.1.2 Raman spectra analysis

**Fig. 3:**
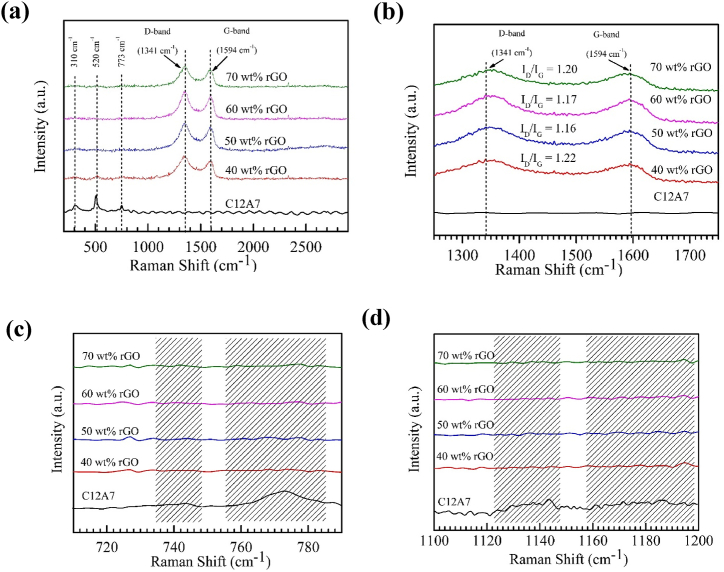
(a) Raman spectra **(b)** Enlargement of the intensity peak in the range of 1250–1750 cm^−1^**(c)** 700–800 cm^−1^, and **(d)** 1100–1200 cm^−1^ of C12A7 and C12A7/rGO composites.

Raman spectra and magnification of the intensity peak in the range of 1250–1750 cm^−1^, 700–800 cm^−1^, and 1100–1200 cm^−1^ of C12A7 and C12A7/rGO composites are illustrated in Fig. 3. According to Fig. 3(a), the Raman shift of C12A7 sample presented characteristic peaks of C12A7 at 310.86, 520.42 and 773.04 cm^−1^ [6,7,10,11]. Raman spectra of all C12A7/rGO composites appear the D-band peak at 1340.90 cm^−1^ and G-band peak at 1594.01 cm^−1^, which corresponding to the translational symmetry of the defect carbon layers (D-band) and the second-order scattering of the sp^2^ carbon (G-band) [8,[12], [13], [14], [15], [16], [17]]. According to Fig. 3(b), the ratio between the intensity of D-peak and the intensity of G-peak (I_D_/I_G_) of C12A7+40 wt%rGO, C12A7+50 wt%rGO, C12A7+60 wt%rGO and C12A7+70 wt%rGO are 1.20, 1.17, 1.16, and 1.22, respectively. These ratio confirm the existence of rGO contents in all C12A7/rGO composite samples [10]. According to Fig. 3(c), the amplification of the intensity peak of C12A7 and all C12A7/rGO composite samples in the range of 700–800 cm^−1^, the Raman shift of C12A7 sample appear two small broad peaks around 740 and 770 cm^−1^. These Raman peaks responsible for $O_{2}^{2-}$ stretching mode of free oxygen ions in the framework of the C12A7 structure [6]. However, the Raman spectra of all C12A7/rGO composite samples not appear any significant intensity peak in the range of 700–800 cm^−1^. According to Fig. 3(d), the amplification of the intensity peak of C12A7 and all C12A7/rGO composite samples in the range of 1100–0.1200 cm^−1^, the Raman shift of C12A7 sample appear two small broad peaks around 1130 and 1175 cm^−1^. These Raman peaks responsible for $O_{2}^{-}$ stretching mode of free oxygen ions in the framework of the C12A7 structure [6]. However, the Raman spectra of all C12A7/rGO composite samples not appear any significant intensity peak in the range of 1100–1200 cm^−1^. Disappearance of Raman intensity peak in the range of 700–800 cm^−1^ and the range of 1100–1200 cm^−1^ of all C12A7/rGO composite samples suggest that the content of rGO in the C12A7/rGO composite cooperating with the heat treatment processes at temperature of 773 K for 30 min under argon atmosphere could reduce the free $O_{2}^{2-}$ and $O_{2}^{-}$ ions from the framework of C12A7/rGO composite [6].

#### 3.1.3 SEM and EDS analysis

**Fig. 4:**
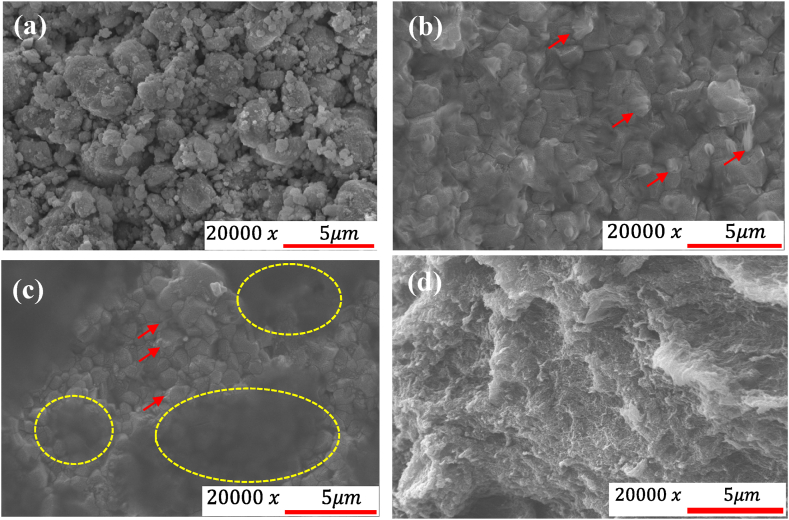
Morphology of **(a)** C12A7 **(b)** C12A7+40 wt% rGO **(c)** C12A7+70 wt% rGO and **(d)** rGO.

The SEM morphology of C12A7, C12A7+40 wt% rGO, C12A7+70 wt% rGO and rGO samples are illustrated in Fig. 4. As can be seen in Fig. 4 (a), the random shape with sharp edge of C12A7 particle was observed [7]. As displayed in Fig. 4 (d), the creases and wrinkles of rGO sheet was observed [11,12]. The grain size of the C12A7 composite is not significantly affected by the loading rGO content. The SEM image of C12A7/rGO composite has been performed to investigate the grain boundaries between the C12A7 matrix and the rGO sheets. According to Fig. 4 (b) and Fig. 4 (c) grain boundaries of C12A7/rGO composite samples after heat treatment at 773 K for 30 min under argon atmosphere were observed. Examples of the homogeneous grain boundary were pointed out by the red arrows. Overall, the homogeneous grain boundary interface in C12A7/rGO composites loading with ultra-high content of rGO presents a promising avenue for optimizing the thermoelectric performance by simultaneously enhancing electrical transport and reducing thermal conductivity. The observation of a homogeneous grain boundary interface in C12A7/rGO composites with loading ultra-high content of rGO is significantly important for their thermoelectric performance. Firstly, the uniform presence of rGO sheets at the grain boundaries creates numerous interfaces enhancing phonon scattering and reducing the lattice thermal conductivity. Secondly, the homogeneous distribution of rGO sheets can facilitate efficient charge carrier transport by acting as conductive pathways, bridging the grain boundaries, and providing continuous electrical connectivity. This can lead to an enhancement in the overall electrical conductivity. Moreover, the homogeneous interfaces minimize the detrimental effects of carrier scattering at the interfaces, maintaining favorable electrical transport properties. Previous research also reports the reduced graphene oxide (RGO) addition effect on the microstructural evolution of the second phases in a series of Sr(Ti, Nb)O_3_ composites leads to improved charge transport within the composite [18]. Fig. 4 (b) and Fig. 4 (c) also display the homogeneous distribution of rGO sheet wrapped on the surface of C12A7 grains. Some rGO sheet were also inserted between the C12A7 grains. Examples of the agglomerated rGO content at grain boundary were pointed out by the red arrows. According to Fig. 4 (c), the opaque area in the SEM image of the C12A7+70 wt% rGO with illustrated by the yellow circle, indicated the rough surface of the multiple layers of the agglomerated rGO sheet.

**Fig. 5:**
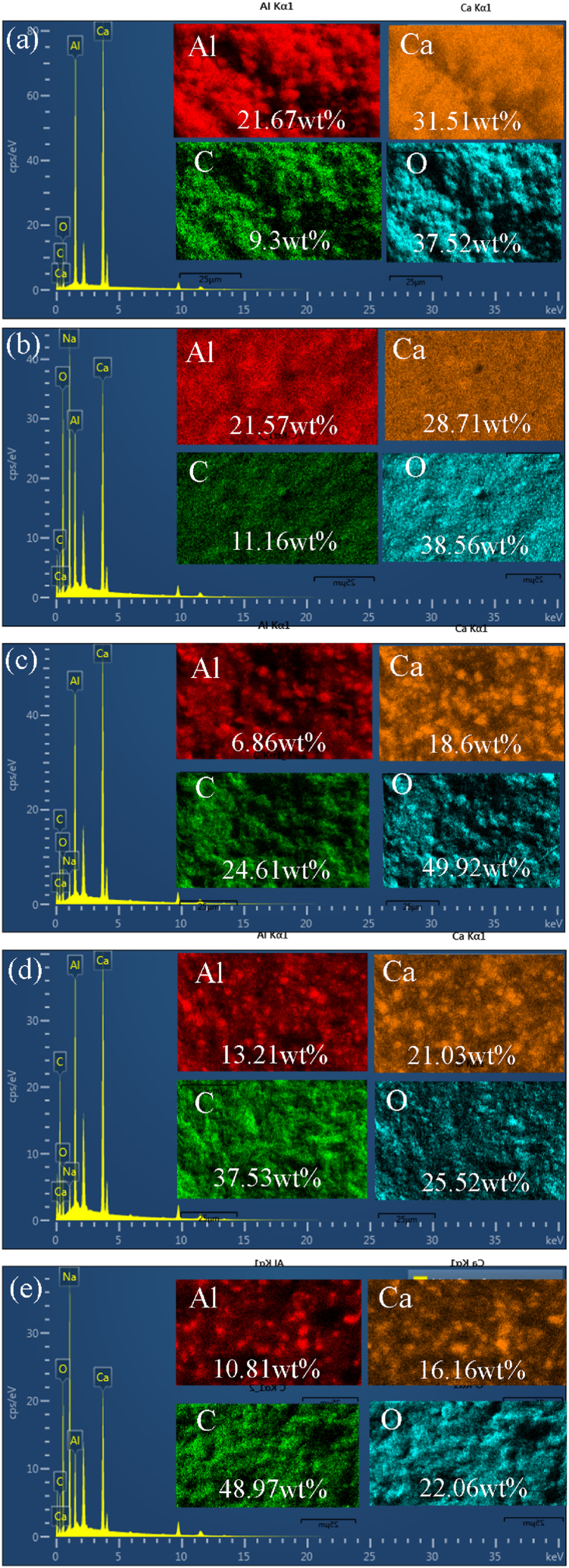
EDS image of **(a)** C12A7 **(b)** C12A7+40 wt% rGO **(c)** C12A7+50 wt% rGO **(d)** C12A7+60 wt% rGO and (e) C12A7+70 wt% rGO samples.

The EDS mapping results of C12A7 and C12A7/rGO composites are illustrated in Fig. 5. The results indicated the O, Ca, Al and C atoms in C12A7 sample and all C12A7/rGO composites samples. These results indicated formation of Ca_12_Al_14_O_33_ phase of ceramic [7]. The results of all C12A7/rGO composites presented the percentage of C atoms was increased with increasing rGO content of the composite. This result indicated existing of the rGO content in all samples after heat treatment at 773 K for 30 min under argon atmosphere.

#### 3.1.4 UV-VIS spectra and optical properties

**Fig. 6:**
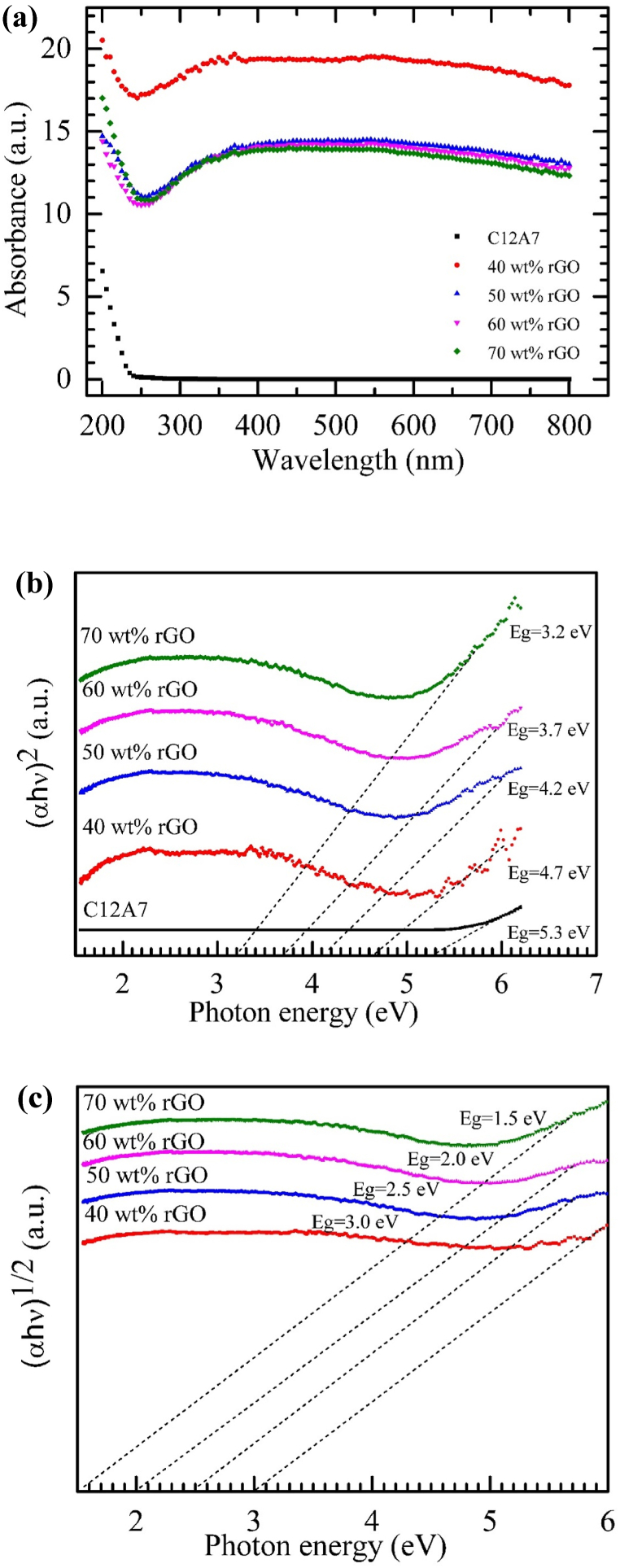
(a) Absorbance **(b)** direct energy gap and **(c)** indirect energy gap of C12A7 and all C12A7/rGO composite samples.

The UV–Visible spectra of C12A7 and all C12A7/rGO composite samples are illustrated in Fig. 6. According to Fig. 6(a), the absorbance spectra from wavelengths of 200–800 nm of C12A7 and all C12A7/rGO composite samples were measured at room temperature. As expected from color of the sample, the absorbance spectra of C12A7 sample (white powder) displayed only absorption peak at wavelength lower than 250 nm. The absorbance spectra of all C12A7/rGO composite samples (black powder) displayed an absorption peak at wavelength lower than 250 nm and another peak at wavelength between 300 and 800 nm. This change in the absorption profile indicates formation of modifications in the energy band structure due to the incorporation of ultra-high amounts of rGO.

The absorbance spectra of all C12A7/rGO composite samples are higher than the absorbance of the C12A7. The absorbance line of C12A7/rGO composite is decreased when the rGO content is increasing. The higher intensity of absorbance may indicate a higher concentration of absorbance species of rGO content or better dispersion of rGO content within the C12A7/rGO composite.

The energy band gap can be calculated from the Tauc plots [19,20] i.e., the relation ${\left(\alpha h\upsilon \right)}^{1/m}=A\left(h\upsilon -E_{g}\right)$, where A is a constant, α is the absorbance, $E_{g}$ is the energy gap and hυ is the photon energy, m=1/2 is the value for the allowed direct band gap and m=2 is the value for the allowed indirect band gap. By plotting the graph between ${\left(\alpha h\upsilon \right)}^{1/m}$ versus hυ, the optical energy gap can be obtained from linearly extrapolating data from the graph to the zero value of the absorption coefficient. According to Fig. 6(b), the direct energy gap can be obtained from the intersection between the linearly extrapolation of the ${\left(\alpha h\upsilon \right)}^{2}$ versus hυ curve and the hυ axis. As illustrated in Fig. 6(b), the calculated direct energy gap of C12A7 sample at room temperature was 5.3 eV. According to Fig. 6(b), the calculated direct energy gap of all C12A7/rGO samples at room temperature was decreased with the content of rGO increasing between 3.2 and 4.7 eV.

According to Fig. 6(c), the indirect energy gap can be obtained from the intersection between the linearly extrapolation of the ${\left(\alpha h\upsilon \right)}^{1/2}$ versus hυ curve and the hυ axis. As illustrated in Fig. 6(c), the calculated indirect energy gap of C12A7 sample at room temperature could not found. According to Fig. 6(c), the calculated direct energy gap of all C12A7/rGO samples at room temperature was decreased with the content of rGO increasing between 1.5 and 3.0 eV.

### 3.2 Electrical transport properties

#### 3.2.1 Electrical conductivity

**Fig. 7:**
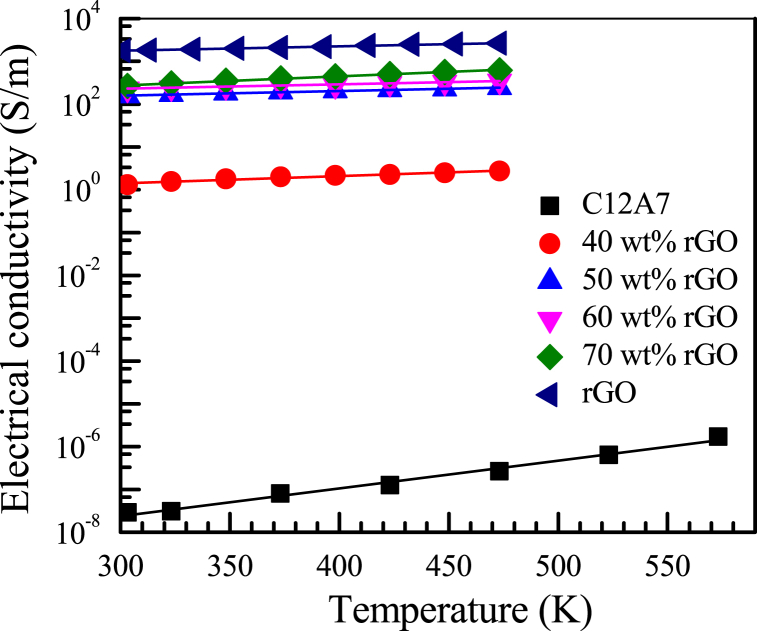
Electrical conductivity of C12A7, rGO and all C12A7/rGO composites.

Electrical conductivity of C12A7, rGO and all C12A7/rGO samples were illustrated in Fig. 7. According to Fig. 7, electrical conductivity between the temperature range of 303–573 K of C12A7 sample displays the lowest value. Without the rGO content, the electrical conductivity of C12A7 was positive temperature dependence between ${10^{-8}-10}^{-6}$ S/m between the temperature range of 303–573 K. The electrical conductivity between ${10^{-8}-10}^{-6}$ S/m during the temperature range of 303–573 K of pristine C12A7 sample after the spark plasma sintering at 1473 K had also been reported by Rudradawong [6]. Between the temperature range of 303–473 K, the electrical conductivity of the rGO sample display the highest value around 62640 S/m and the electrical conductivity of all the ultra-high amount C12A7/rGO composite samples were increased with the content of rGO increasing between 2.7−620 S/m. The electrical conductivity of rGO sample and all C12A7/rGO composite sample were temperature independence. According to Rudrarawong [6], The electrical conductivity of the mayenite ceramic and carbon black (C12A7/nCB) composites (3,5 and 10 wt%) had been reported temperature independence during the temperature range of 300–1073 K and increased between 37 and 356 S/m with the nCB content increasing. Unfortunately, the electrical conductivity of 0 and 1 wt% of C12A7/nCB composites had been reported temperature dependence and increased with the nCB content increasing. These results indicated specific behavior of the C12A7 composite with the difference amount of second phase materials.

Several mechanisms are responsible for the significant improvement in electrical conductivity of C12A7/rGO composites with ultra-high rGO loading. Firstly, the rGO sheets can form an interconnected network within the C12A7 matrix, providing efficient pathways for charge carrier transport. This network facilitates the flow of electrons through the highly conductive rGO sheets, significantly enhancing the overall electrical conductivity of the composite. Secondly, the interaction between the rGO sheets and the C12A7 matrix can result in charge transfer or hybridization of the electronic states, modifying the energy band structure and increasing the density of states near the Fermi level. The introduction of the electronic states near the Fermi level can facilitate easier charge carrier excitation and transport, contributing to the improvement in electrical conductivity.

#### 3.2.2 Carrier concentration

**Fig. 8:**
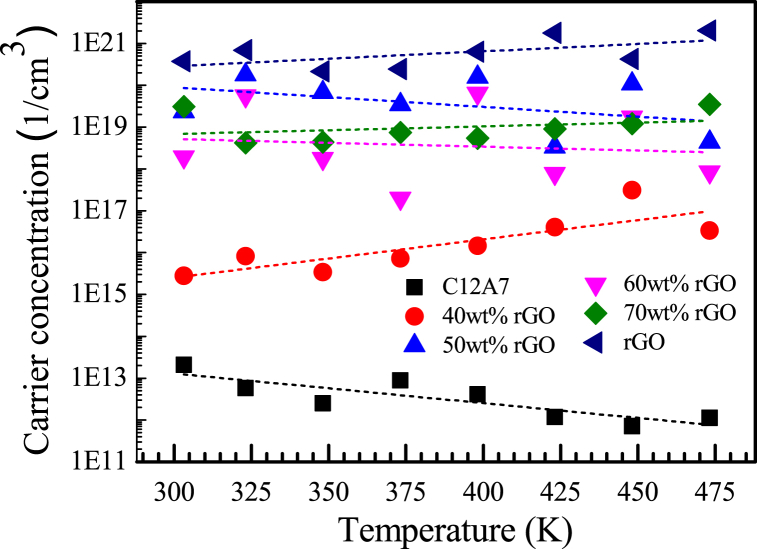
Carrier concentration of C12A7, rGO and C12A7/rGO composite samples.

Carrier concentration of C12A7, rGO and C12A7/rGO composite samples between the temperature range of 303–473 K were shown in Fig. 8. The carrier concentration of C12A7 sample display the lowest value between ${10^{11}-10}^{13}$ S/cm^−3^. The carrier concentration of rGO sample display the highest value between ${10^{20}-10}^{22}$ S/cm^−3^. The carrier concentration of the C12A7+40 wt% rGO samples display the value between ${10^{15}-10}^{17}$ S/cm^−3^. The carrier concentration of the C12A7+50 wt% rGO, C12A7+60 wt% rGO, and C12A7+70 wt% rGO samples display higher values between ${10^{17}-10}^{21}$ S/cm^−3^. The incorporation of ultra-high amounts of rGO can potentially increase the carrier concentration within the composite by providing additional charge carriers or introducing defects that act as dopants. A higher carrier concentration can lead to an increase in electrical conductivity.

#### 3.2.3 Mobility

**Fig. 9:**
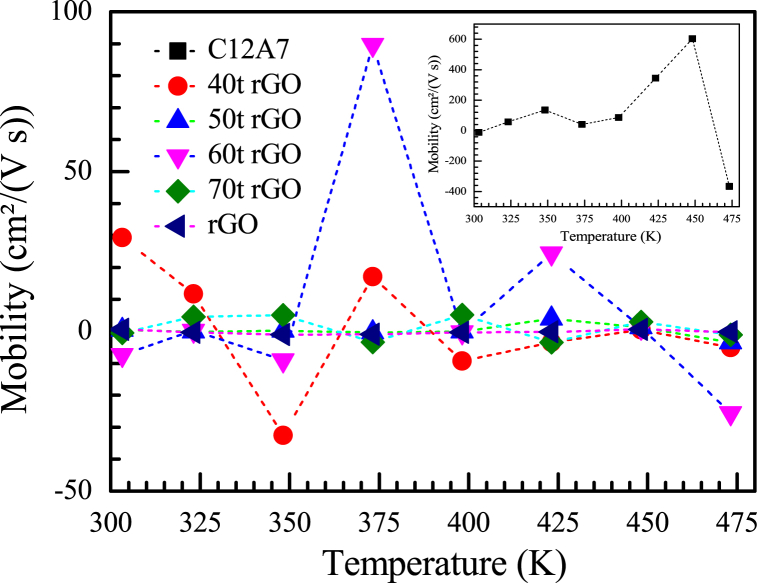
Mobility of C12A7, rGO and C12A7/rGO composite samples.

Mobility of C12A7, rGO and C12A7/rGO composite samples between the temperature range of 303–473 K were shown in Fig. 9. According to Fig. 9, while the mobility of C12A7 sample was the less stable value between −400 and 600 cm^2^/Vs, the mobility of rGO sample was the most stable value around zero cm^2^/Vs with respect to temperature.

### 3.3 Thermoelectric properties

#### 3.3.1 Seebeck coefficient

**Fig. 10:**
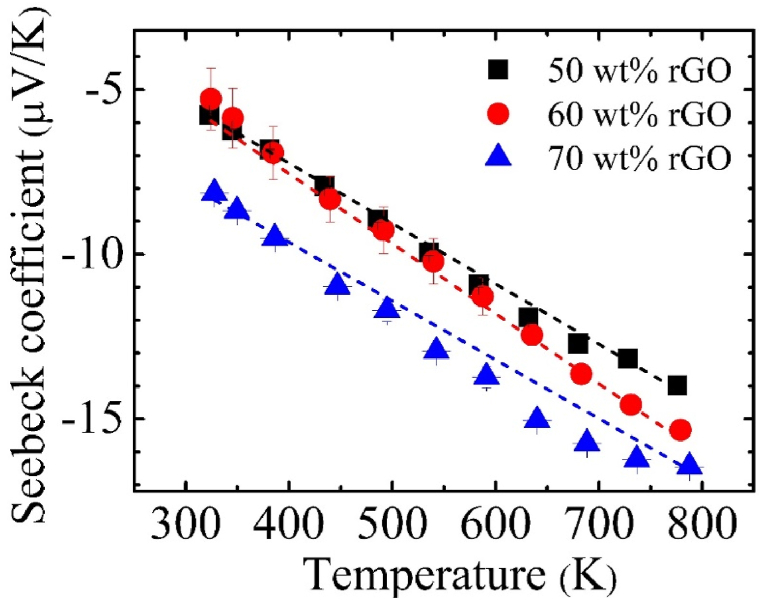
Seebeck coefficient of C12A7/rGO composite samples.

Seebeck coefficient of C12A7+50 wt% rGO, C12A7+60 wt% rGO, and C12A7+70 wt% rGO between the temperature range of 303–800 K were illustrated in Fig. 10. According to Fig. 10, Seebeck coefficient of all C12A7/rGO composite samples were negative sign and temperature dependence between the range of −5 to −17 μV/K. Absolute value of the Seebeck coefficient of all C12A7/rGO composite samples were increased when rGO content increasing. Seebeck coefficient of C12A7/rGO composites loading with ultra-high content of rGO exhibits a temperature dependence, increasing with temperature. The presence of ultra-high amounts of rGO of C12A7/rGO composites can modify the energy band structure and carrier concentration, potentially affecting the temperature dependence of the Seebeck coefficient. The Seebeck coefficient of the 3, 5 and 10 wt% of C12A7/nCB composites during the temperature range of 300–950 K had been reported by Rudradawong [6]. The results display positive sign, temperature dependence and increased between 0 and 25 μV/K with the second phase material (nCB) content increasing.

#### 3.3.2 Power factor

**Fig. 11:**
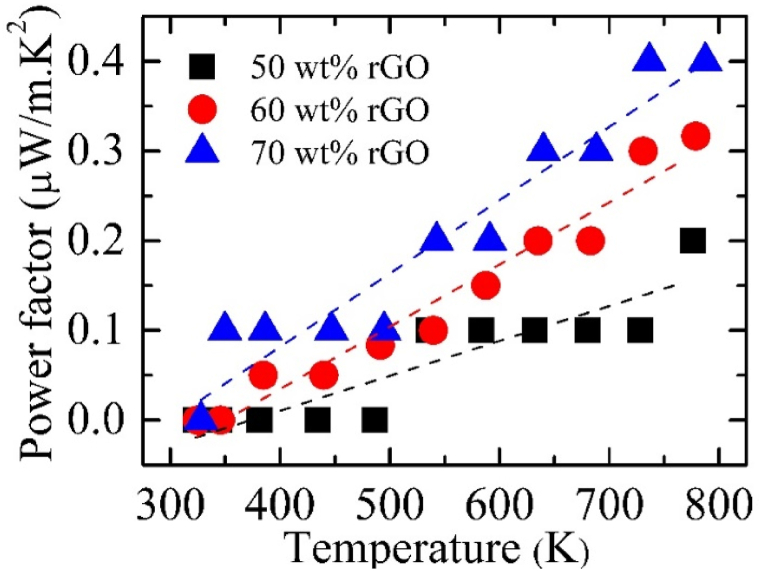
Power factor of C12A7/rGO composite samples.

Power factor of C12A7+50 wt% rGO, C12A7+60 wt% rGO, and C12A7+70 wt% rGO between the temperature range of 303–800 K were illustrated in Fig. 11. According to Fig. 11, the PF of all C12A7/rGO composite samples was temperature-dependent within the range of 0.4 $\mu W/m.K^{2}$. The power factor of all C12A7/rGO composite samples was increased with rGO content increasing.

#### 3.3.3 Thermal conductivity

**Fig. 12:**
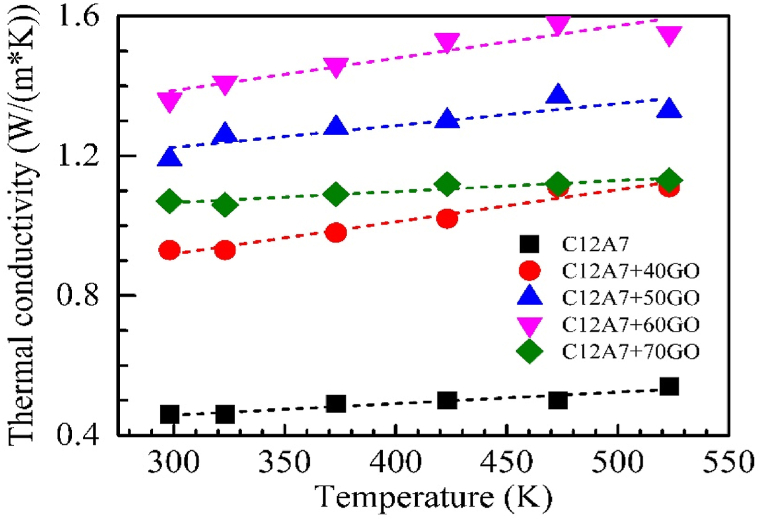
Thermal conductivity of C12A7 and C12A7/rGO composite samples.

Thermal conductivity of C12A7 sample and all C12A7/rGO composite samples between the temperature range of 303–800 K were illustrated in Fig. 12. According to Fig. 12, the thermal conductivity of C12A7 sample and all C12A7/rGO composite samples were slightly temperature dependence and increased with the rGO content increasing.

#### 3.3.4 Dimensionless figure of merit (ZT)

**Fig. 13:**
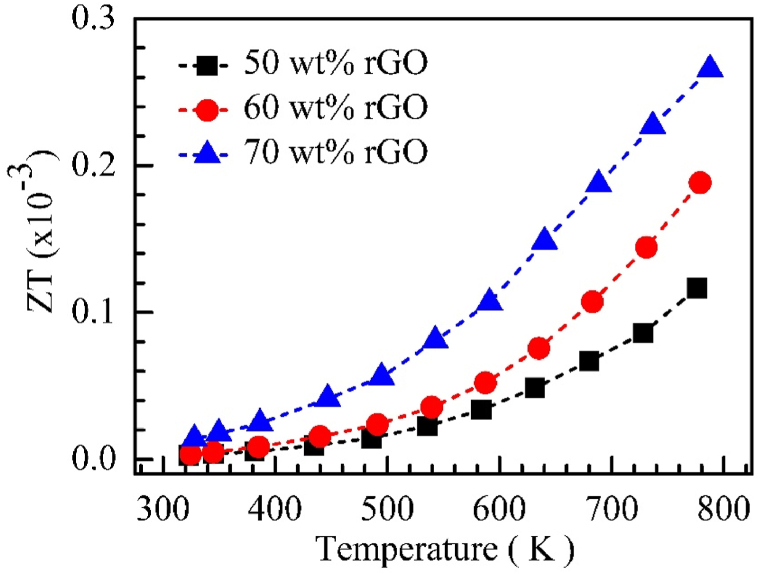
ZT of C12A7/rGO composite samples.

Dimensionless figure of merit (ZT) of C12A7+50 wt% rGO, C12A7+60 wt% rGO, and C12A7+70 wt% rGO between the temperature range of 303–800 K were illustrated in Fig. 13. According to Fig. 13, the ZT of all C12A7/rGO composite samples were positive temperature-dependent within the range of ${3x10}^{-4}.\;\text{The}\;\text{ZT}$ of all C12A7/rGO composite samples was increased with rGO content increasing.

### 3.4 Energy gap and thermoelectric properties of the C12A7/rGO composite

The C12A7/rGO composites with ultra-high rGO loading result in a narrowing of the direct energy band gap and the creation of indirect energy band gap. The electrical conductivity of the ultra-high amount C12A7/rGO composites were significantly improved with respect to the C12A7 sample. The electrical conductivity also increased with the content of rGO increasing. By incorporating ultra-high amounts of rGO, the composite can benefit from the high conductivity of rGO. The conductive rGO content provides network for charge carrier transport, leading to an increase in the electrical conductivity and the carrier concentration of the C12A7/rGO composites. According to the Mott formula, the Seebeck coefficient is proportional to the energy derivative of the density of states at the Fermi level. The high concentration of rGO can introduce additional electronic states near the Fermi level and improve the Seebeck coefficient of the C12A7/rGO composite. At ultra-high loadings, the rGO network can contribute to an increase in the overall thermal conductivity of the C12A7/rGO composite. However, the incorporation of ultra-high rGO into C12A7 can lead to the formation of numerous interfaces and grain boundaries between the rGO sheets and the C12A7 matrix. These interfaces and grain boundaries can act as effective scattering centers for phonons, reducing the lattice thermal conductivity. The C12A7 matrix plays a crucial role in determining the thermoelectric properties of C12A7/rGO composites with ultra-high rGO loading. This influence is due to three key factors. Firstly, rGO and C12A7 interact and modify the overall band gap of the composite. Secondly, charge transfer or hybridization of electronic states between the rGO sheets and the C12A7 matrix can modify the density of states near the Fermi level, impacting both the Seebeck coefficient and electrical conductivity. Finally, a homogeneous grain boundary interface between the rGO sheets and the C12A7 matrix grains allows phonon scattering at these interfaces and grain boundaries, ultimately reducing the lattice thermal conductivity of the composite. To achieve a high figure of merit (ZT), it is essential to maximize power factor and minimize thermal conductivity of the C12A7/rGO composite.

## 4 Conclusions

The C12A7 and ultra-high amount i.e., 40, 50, 60 and 70 wt% of rGO composites were synthesized by solid state reaction process, then the heat treatment with the temperature range of 773 K for 30 min under argon atmosphere was applied to the samples pellet. Existing of the rGO content in all C12A7/rGO composite sample was confirmed by XRD, Raman, SEM image, EDS mapping. The grain boundary of the C12A7/rGO composite samples after the heat treatment, homogeneous wrapped structure of C12A7 grain by rGO sheet, and agglomerated rGO contents were observed from SEM images. The optical properties of the C12A7/rGO composites were investigated by UV-VIS. The direct and indirect energy gap of the C12A7/rGO composite samples were calculated from the absorbance spectra of the samples. Both calculated direct and indirect gap predicted decreased energy gap when the rGO content increasing. Electrical conductivity, carrier concentration and mobility of C12A7/rGO composite samples were investigated using Hall characterization system. The results indicated increased of electrical conductivity and carrier concentration when the content of rGO increasing. The Seebeck coefficient of C12A7/rGO composite samples indicated n-type properties of the composites. The Seebeck coefficient of C12A7/rGO composite samples also temperature dependence and the absolute value were increased when the rGO content increasing. The thermal conductivity of C12A7/rGO composite samples were increased with the rGO content increasing. Power factor and dimensionless figure merit of C12A7/rGO composite samples were increased with the rGO content increasing.

The observed improvements in electrical and thermoelectric properties of C12A7/rGO composites with ultra-high rGO loadings have several practical implications. The improved electrical conductivity and optimized Seebeck coefficient in the C12A7/rGO composites can lead to a higher power factor and figure of merit. A higher ZT translates to better energy conversion efficiency. The networks of rGO sheets and charge transfer effects can result in higher power output from thermoelectric devices based on these composites. This increased power output can be beneficial for applications requiring higher energy densities, such as powering electronic devices or supplying power to remote or off-grid locations. The potential for reduced thermal conductivity through efficient phonon scattering at grain boundaries and interfaces minimizes heat dissipation, allowing for smaller and more efficient designs. The ability to tailor the microstructure, composition, and energy band structure of C12A7/rGO composites through interface engineering, grain boundary engineering, and rGO dispersion optimization offers versatility in material design. The use of inexpensive C12A7 as a constituent material in C12A7/rGO composites offers advantages in terms of scalability and cost-effectiveness. While the use of ultra-high amounts of rGO from graphite increases the economic viability of rGO and these composites. Thermoelectric devices based on C12A7/rGO composites can contribute to environmental sustainability by enabling waste heat recovery and energy harvesting.

## CRediT authorship contribution statement

**Keerati Maneesai:** Writing – review & editing, Writing – original draft, Visualization, Data curation, Formal analysis, Investigation, Methodology, Resources, Validation. **Montree Thongkam:** Resources. **Chaval Sriwong:** Writing – original draft, Visualization, Validation, Supervision, Resources, Methodology, Formal analysis, Data curation, Writing – review & editing. **Chesta Ruttanapun:** Project administration, Methodology, Investigation, Funding acquisition, Formal analysis, Data curation, Validation, Writing – original draft, Writing – review & editing, Supervision, Conceptualization.

## Declaration of competing interest

The authors declare that they have no known competing financial interests or personal relationships that could have appeared to influence the work reported in this paper.
